# Hybrid Procedures. Opening Doors for Surgeon and Cardiologist Close Collaboration

**DOI:** 10.3389/fped.2021.687909

**Published:** 2021-07-27

**Authors:** Juan-Miguel Gil-Jaurena, José-Luis Zunzunegui, Ramón Pérez-Caballero, Ana Pita, Carlos Pardo, Corazón Calle, Uxue Murgoitio, Fernando Ballesteros, Alejandro Rodríguez, Constancio Medrano

**Affiliations:** ^1^Pediatric Cardiac Surgery, Hospital Gregorio Marañón, Madrid, Spain; ^2^Instituto de Investigación Sanitaria Gregorio Marañón, Madrid, Spain; ^3^Pediatric Cardiology, Hospital Gregorio Marañón, Madrid, Spain

**Keywords:** hybrid, univentricular, biventricular, transplant, cardiologist, surgeon

## Abstract

**Background:** Collaboration between cardiac surgeons and cardiologists can offer interventions that each specialist may not be able to offer on their own. This type of collaboration has been demonstrated with the hybrid Stage I in patients with hypoplastic heart syndrome. Since that time, a hybrid approach to cardiac interventions has been expanded to an incredible variety of potential indications.

**Methods:** Seventy-one patients were scheduled for a hybrid procedure along 8 years. This was defined as close collaboration between surgeon and cardiologist working together in the same room, either cath-lab (27 patients) or theater (44 patients).

**Results:** Six groups were arbitrarily defined. A: vascular cut-down in the cath-lab (27 neonates); B: bilateral banding (plus ductal stent) in hypoplastic left heart syndrome or alike (15 children); C: perventricular closure of muscular ventricular septal defect (10 cases); D: balloon/stenting of pulmonary branches along with major surgical procedure (12 kids); E: surgical implantation of Melody valve (six patients) and others (F, one case). Two complications were recorded: left ventricular free wall puncture and previous conduit tearing. Both drawbacks were successfully sort out under cardiopulmonary by-pass.

**Conclusion:** Surgeon and cardiologist partnership can succeed where their isolated endeavors are not enough. Hybrid procedures keep on spreading, overcoming initial expectations. As a bridge to biventricular repair or transplant, bilateral banding plus ductal stent sounds interesting. Novel indications can be classified into different groups. Hybrid procedures are not complication-free.

## Background

Surgical and percutaneous procedures have experienced striking advances in congenital heart diseases along the past decades. Collaboration between interventional cardiologists and cardiac surgeons has recently paved the way for the so-called “Hybrid procedures”. In offering a hand-to-hand intervention, the result becomes less invasive and more efficient, rendering a safer management for the congenital condition.

Partnership in the field of aortic valve replacement in the adult population (either trans-femoral or trans-apically deployed) is an example of hybrid approach. The first report of a hybrid procedure (1) in a neonate with hypoplastic left heart syndrome was launched in 1993. A surgical bilateral pulmonary banding followed by a percutaneous ductal stenting was performed. But it was in 2004 when the first single-room hybrid approach (2) took place, actually. Rather than the milestone itself, the importance relies in the new concept of collaborative approach in complex congenital heart diseases, which often require several open heart surgeries throughout their lives well beyond adulthood. Novel hybrid procedures have the goal of reducing the total number of invasive surgeries over a lifetime span and reducing morbidity and mortality of certain interventions.

This holds true not only for the hypoplastic left heart pathway but also for a vast array of cardiac conditions in which surgical or percutaneous procedures in isolation cannot address the problem. As this field keeps on growing, more sophisticated hybrid rooms will be devised to cope with the increasing needs of both surgeons and cardiologists.

Our group experience along eight consecutive years is presented. Provided the sparking use and indications for hybrid procedures, we intend to cluster them into several categories. The rationale for assignment to cath-lab or theater will be discussed and the complications analyzed.

## Methods

From January 2013 until December 2020, 71 patients underwent a hybrid procedure to treat their cardiac condition in our Institution. The studies involving human participants were reviewed and approved by Hospital Gregorio Marañón's ethics committee. The patients/participants parents provided written informed consent to participate in this study. Data are recorded in a prospective manner. According to underlying disease and main procedure accomplished, they have been arbitrarily divided into groups (Table 1):

A. Cut-down vascular access (27 patients)
B. Hypoplastic left heart syndrome or alike (15 children)
C. Perventricular ventricular septal defect closure (10 cases)
D. Pulmonary artery branches dilatation/stenting (along with surgical repair; 12 infants)
E. Biological valve (Melody) deployment in open-heart field (6 patients)
F. Others (one case).

**Table 1:** Distribution of hybrid procedures in clusters.

**Vascular access (27)**	**Bi-banding + ductal stent (15)**	**Muscular VSD (10)**	**Angio/stent branches (12)**	**Surgical Melody (6)**	**Others (1)**
**Cath-lab: 27: Theater: none**	**Cath-lab: 10 Theater: 5**	**Cath-lab: none** ^*^ **Theater: 10**	**Cath-lab: none Theater: 12**	**Cath-lab: none Theater: 6**	**Cath-lab: 1**
Carotid cut-down (25)	Comprehensive (3)	Apical VSD (2)	Pulm prosthesis after Fallot (3)	Mitral (2)	Pulmonary vein stenosis (1)
Yugular cut-down (2)	Biventricular (4)	no CPB (4)	Conduit replacement (3)	Tricuspid (1)	
	Transplant (7)	with CPB (4)	Fontan completion (1)	Off-label pulmonary (2)	
	Died in transplant list (1)		“second” hybrid (5)	Sub-xyphoid conduit replacement (1)	

*VSD, ventricular septal defect. CPB, cardiopulmonary by-pass. Twenty-seven procedures were performed in the cath-lab and 44, in theater*.

(*) *: Two patients were first taken to the cath-lab and, then, to the Table 2. Bridge to uni-, bi-ventricular, or transplant pathways in HLHS (or alike) with hybrid pulmonary stenting plus bilateral banding*.

The decision to carry out the hybrid procedure in the cath-lab or in surgical theater was based upon the likelihood of cardiopulmonary by-pass needs (performed in theater). Cath-lab and theater are located in the same level, with a corridor in between both suites. Transition from cath-lab to theater, or the other way round, is feasible (distinguished as two-stage hybrid procedure).

Upon this rationale, cut-downs and hypoplastic left heart syndrome hybrid approaches (groups 1 and 2) were mainly performed in the cath-lab, whereas most ventricular septal defect closure, pulmonary branches balloon dilatation, and Melody insertion took place in theater (groups 3, 4, and 5 were scheduled on cardiopulmonary by-pass).

Technique:

1. **Vascular access in the cath-lab**. Particularly for neonates and infants below 5 kg requiring aortic valvuloplasty. An incision is performed in the neck by the surgeon. The carotid artery is dissected and looped before purse-string or cut-down cannulation (3). Should Extra Corporeal Membrane Oxygenation (ECMO) is needed, the jugular vein is easily available. Upon finishing the procedure, the surgeon sutures the vessel and checks its patency (4).
2. **HLHS**. For those patients with a hypoplastic left heart syndrome, or alike, and not amenable to Norwood I procedure because a high risk was anticipated, a single-stage hybrid procedure in the cath-lab was offered. Bilateral banding (Doppler velocity around 4 m/sec) plus open cell stent deployment through the entire length of the ductus was performed (5). Balloon septostomy/stent was deferred as a separate procedure, if requested (6). In other group of patients, bilateral banding plus ductal stent was applied as a bridge to transplant, or palliation in some complex forms of left heart obstructive lesions, in order to promote chances of biventricular repair (7, 8).
3. **Muscular ventricular septal defect**. Through mini-sternotomy approach, under echo guidance, the right ventricle free wall is depressed with the tip of the forceps just opposite to the muscular septal defect and a purse-string is secured on a tourniquet. Using Seldinger technique, the free wall is punctured with a needle, followed by a guide wire and a sheath. A device with a waist 2 mm larger than the septal defect diastolic diameter is chosen. The left disk is opened, then pulled against the septum to fit the waist in the defect, and followed by the right disk deployment (9). Tricuspid and aortic valves are tested for regurgitation, and gross residual shunts are ruled out before device release. Full sternotomy and cardiopulmonary by-pass is chosen when concomitant procedures are requested (debanding, atrial septal defect closure, etc.) For apical muscular defects, patients have a soft guide/balloon inserted in the septal defect *via* transfemoral access in the cath-lab. Then, they are driven to theater and, under cardiopulmonary bypass, a tiny right apical ventriculotomy is performed to identify the guide/balloon. After trimming the trabeculae around the guide, the ventricular septal defect edges are identified and eventually closed.
4. **Balloon dilatation/stenting in pulmonary arteries**. Performed with the need for a concomitant surgical procedure (10) such as conduit replacement, pulmonary valve implantation, ventricular septal defect closure, etc. It involves dilatation/stenting under direct vision during open heart surgery, while on cardiopulmonary bypass, in a beating heart. Stents can be manually flared against the wall of the main pulmonary artery to make future access to the vessel easier, and sutures can be placed in the proximal edge of the stent to prevent distal embolization (11).
5. **Melody deployment** in pulmonary position through a subxyphoid approach (12). Inasmuch the Melody valve was initially approved for the pulmonary position only, delivery in mitral (13) location was reported in 2014. The device is flared, so as not to obstruct the left ventricle, and sutured in the mitral annulus. After securing it, the valve is balloon dilated to the target diameter upon direct vision. Further percutaneous dilatations can be scheduled as the child grows.
6. **Others**. Pulmonary vein stenosis. A midline sternotomy is fashioned and a purse-string suture is placed in the right superior pulmonary vein. After introducing a soft wire, a stent is deployed in the opposite left pulmonary veins to relieve their narrowing (14).

## Results

**Figure 1:**
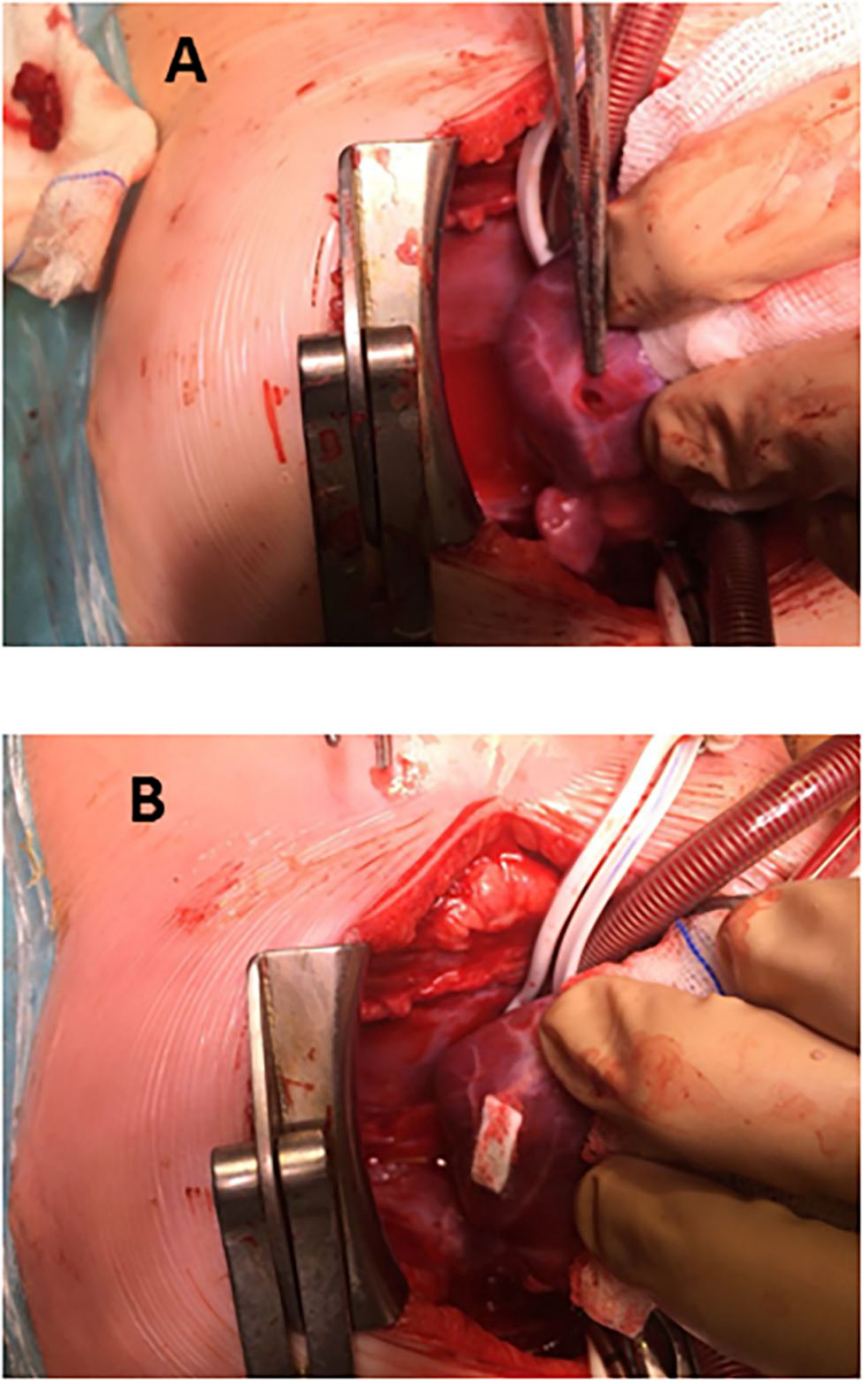
Perventricular closure of muscular ventricular septal defect. **(A)** Accidental puncture in lateral wall of the left ventricle, close to a marginal coronary artery. **(B)** Glued-patch repair of fee-wall puncture.

**Figure 2:**
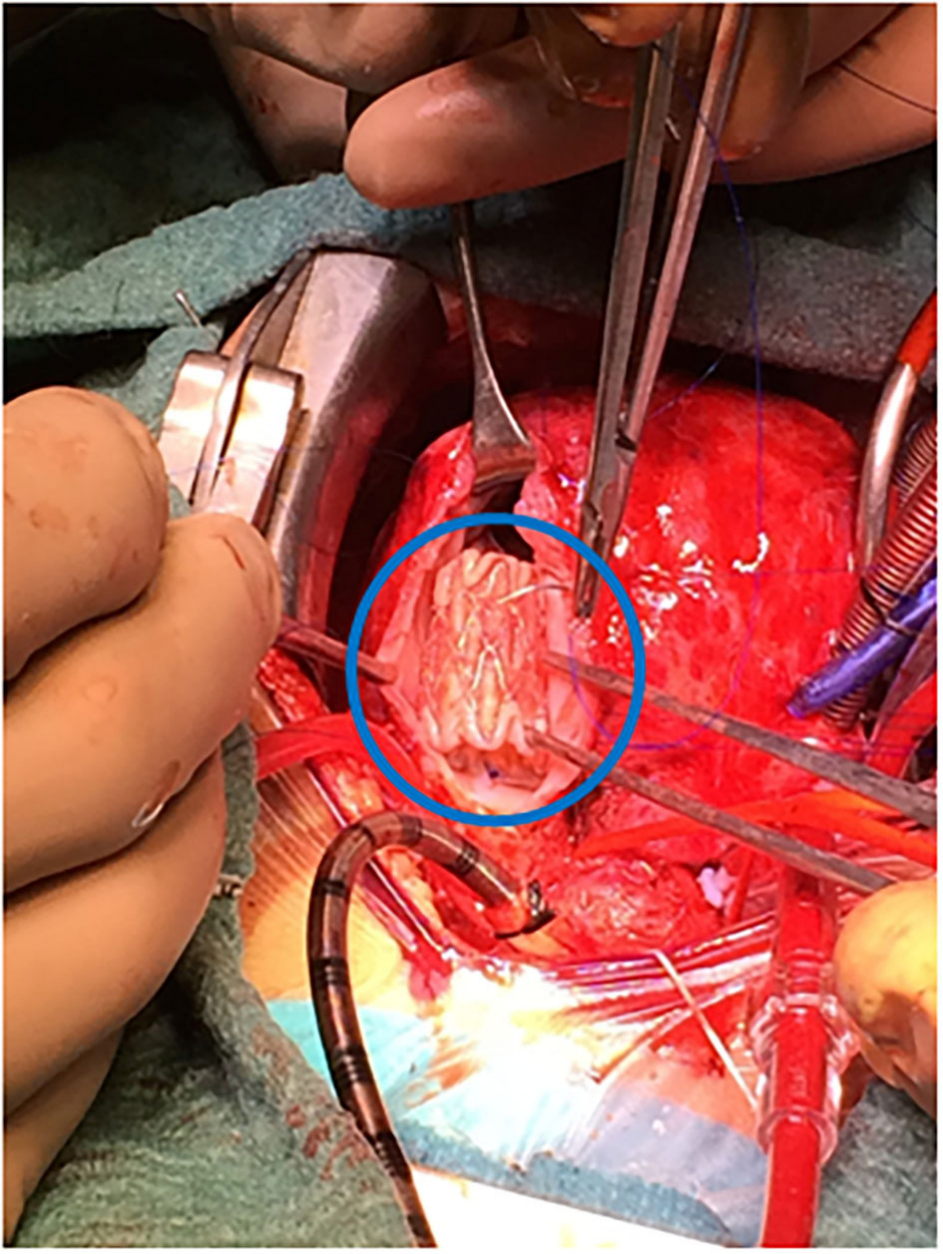
Surgical insertion of melody valve (blue encircled) in an arrested heart on cardiopulmonary bypass.

A. Cut-down vascular access. All cases (27) took place in the cath-lab. Carotid artery was approached in 25 neonates for aortic valvuloplasty (22 children), ductal stent (one Fallot), coarctation angioplasty (one patient), and coarctation stent (one case). The jugular vein was used twice: in a 4-month-old Fallot patient on transthoracic ECMO for stenting the right ventricular outflow tract and in a 2-year-old infant with a previous Melody in the tricuspid position (which happened to be regurgitant) in whom a valve-in-valve procedure was attempted. On completing the procedure, the vessel was repaired (either stitching the cut-down or closing the purse-string suture) and the patency checked.
B. Hypoplastic left heart syndrome, or alike (15 patients, Table 2)
  - Ten patients were treated in the cath-lab and five in theater (after decision change). Six neonates with hypoplastic left heart syndrome were deemed unsuitable for conventional Norwood I technique because of low weight (1.8 kg), myocardial dysfunction, severe tricuspid regurgitation, or ECMO resuscitation. In the same period of time, nine classical Norwood were carried out (with two deaths).
  - Four patients were “bridged” to promote bi-ventricular repair, either as an intention-to-treat (two cases) or after a decision-making in theater (two children, who left the operating-room with a bilateral banding in a “bridge to decision” strategy and had their ductus stented in the cath-lab as a two-stage procedure).
  - Seven patients underwent subsequent atrial septostomy plus stent deployment. Another child had to be re-banded.
  - At follow-up, four patients had a bi-ventricular repair (2 Ross-Konno, 1 Yasui-Norwood plus Rastelli, and 1 arch repair plus ventricular septal defect closure), seven were transplanted (15, 16), and three underwent a comprehensive Norwood plus Glenn repair. One patient died in the transplant waiting list.
  - Eventually, four out of the 10 patients died, one in each group: arch repair (ventricular arrhythmia), one transplant (pneumonia), one comprehensive repair (pulmonary hypertension), and the abovementioned in the transplant waiting list.
C. Muscular ventricular septal defect (10 cases, depicted in Table 3).
  - Two patients had their apical muscular septal defect closed by the hybrid two-stage procedure: first, marking it in cath-lab (apical septal defect location) and then driven to theater (for closure under cardiopulmonary bypass) with good result (trivial residual ventricular septal defect).
  - Eight children had their muscular ventricular septal defect closed by a per-ventricular approach.
    - In four cases, provided that it was a single defect, a mini-sternotomy without cardiopulmonary by-pass was scheduled.
    - The four remaining ones were approached through a full-sternotomy and cardiopulmonary by-pass, since other defects had to be addressed: patching of a common atrium, perimembranous ventricular septal defect, debanding, and aortic arch repair, respectively. One of the patients (actually, the first one in our series) was complicated by a puncture in the left ventricle lateral wall (Figure 1A). Because the pump machine was on back-up for the larger perimembranous ventricular septal defect closure, we run the cardiopulmonary by-pass and on the empty beating heart a glued-patch was gently applied (Figure 1B) before proceeding with a routinely trans-tricuspideal both perimembranous and muscular septal defects closure.
  154 VSD were operated on in the same lapse of time.
D. Surgical dilatation of pulmonary branches (12 children). Concomitant surgical repair and pulmonary arteries dilatation was accomplished in 12 children.
  - Three former Fallot patients had their left pulmonary branch balloon-dilated and then a biological pulmonary valve implanted on a beating-heart basis.
  - Three children who underwent right ventricle to pulmonary artery conduit replacement (2 Rastelli, 1 Ross-Konno) had their left pulmonary branch balloon-dilated (2) or stented (1) in the same fashion.
  - One patient had a plug delivered in the pulmonary trunk by a perventricular puncture along with Fontan completion.
  - Five patients with a previous “hybrid bilateral banding” had their pulmonary arteries revisited: a 3.4 kg transplant who suffered a tear in the right pulmonary artery and had a covered stent deployed, two more transplant children, and two patients (arch repair and Yasui) who were electively scheduled for branches dilatation at the time of band removal plus bi-ventricular repair.
E. Melody deployment in theater (6 cases).
  - Mitral regurgitation after a switch procedure in a neonate was unsuccessfully addressed by artificial chordal insertion and eventually treated with a Melody under cardiopulmonary by-pass and cardioplegic arrest. Unfortunately, the child died on septicemia several days later, with a well functioning valve. A second patient was successfully operated on under the same strategy.
  - A neonate with pulmonary atresia and intact ventricular septum plus severe tricuspid regurgitation had a Melody implanted in tricuspid position, after a failed attempt of valve repair (plus conduit insertion between right ventricle and pulmonary artery).
  - Two former Fallot patients presenting with residual VSD and pulmonary regurgitation had the ventricular septal defect addressed, plus a transannular Melody valve implanted (as a biological valve insertion) and balloon dilated in the pulmonary position (Figure 2).
  - An infant with a right ventricle to pulmonary conduit in place was scheduled for a perventricular conduit deployment by sub-xyphoideal approach. One purse-string was stitched in the diaphragmatic right ventricle wall. The stiff wire happened to dissect the previous conduit and emergent re-sternotomy plus cardiopulmonary bypass was instituted, ending up in a surgical conduit replacement.
  - 121 percutaneous and 73 surgical pulmonary valves were implanted between 2013 and 2020 by our group.
F. Pulmonary vein stenosis. A 5-month-old infant with congenital left pulmonary vein stenosis was attempted to treat percutaneously twice. Transeptal approach proved unsuccessful. In the cath-lab, by midline sternotomy, a purse-string suture was fashioned in the right superior pulmonary vein (as for a regular “vent” sucker in theater) and a sheath advanced through it. Then, a *BUS* absorb stent was deployed in the opposite left superior pulmonary vein under fluoroscopy.

**Table 2:** Bridge to uni-, bi-ventricular or transplant pathways in HLHS (or alike) with hybrid pulmonary stenting plus bilateral banding.

** 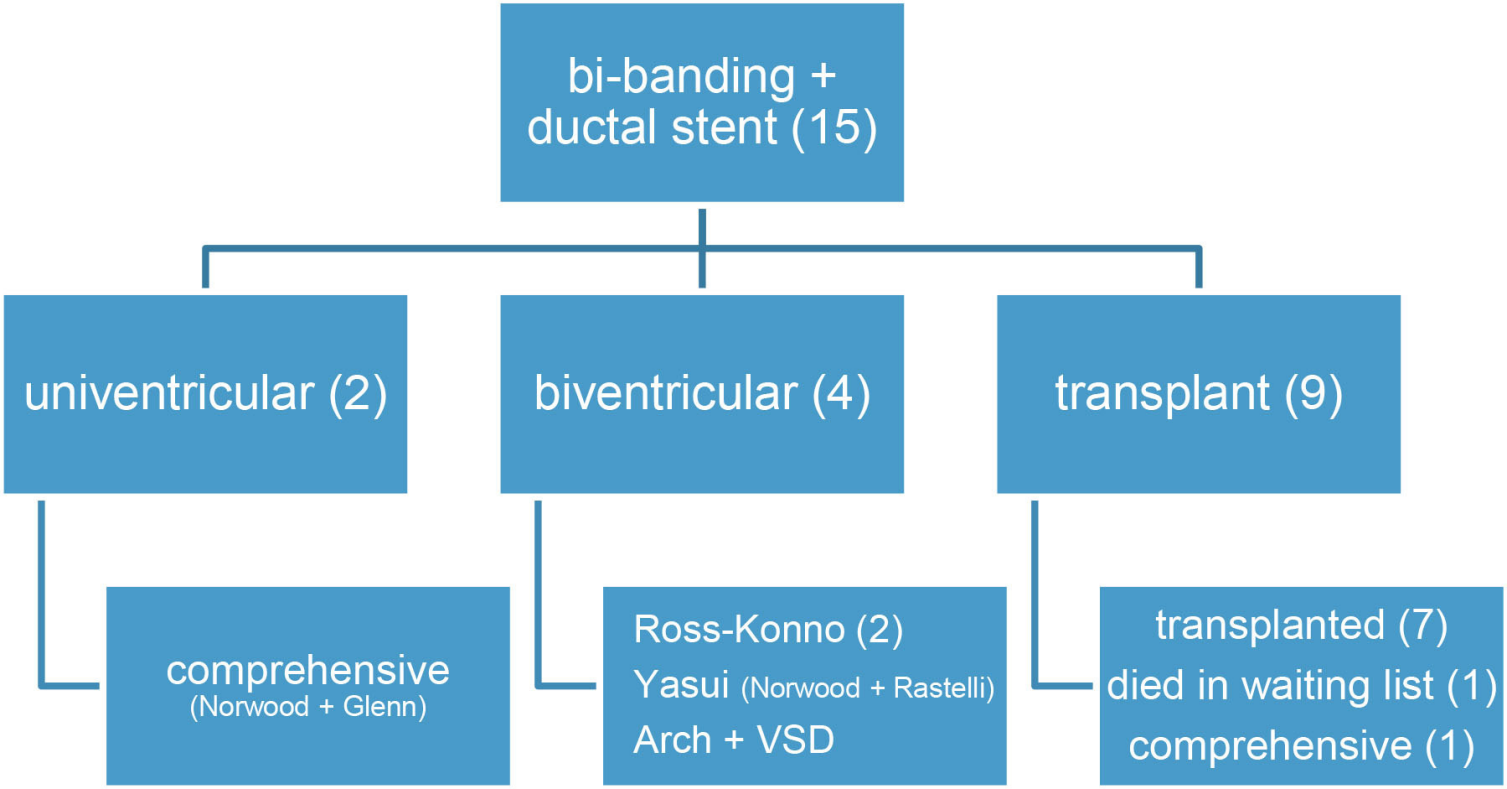 **

**Table 3:** Hybrid approach for muscular VSD (ventricular septal defect). ASD, atrial septal defect. CPB, cardiopulmonary by-pass. Pm, perimembranous (VSD).

** 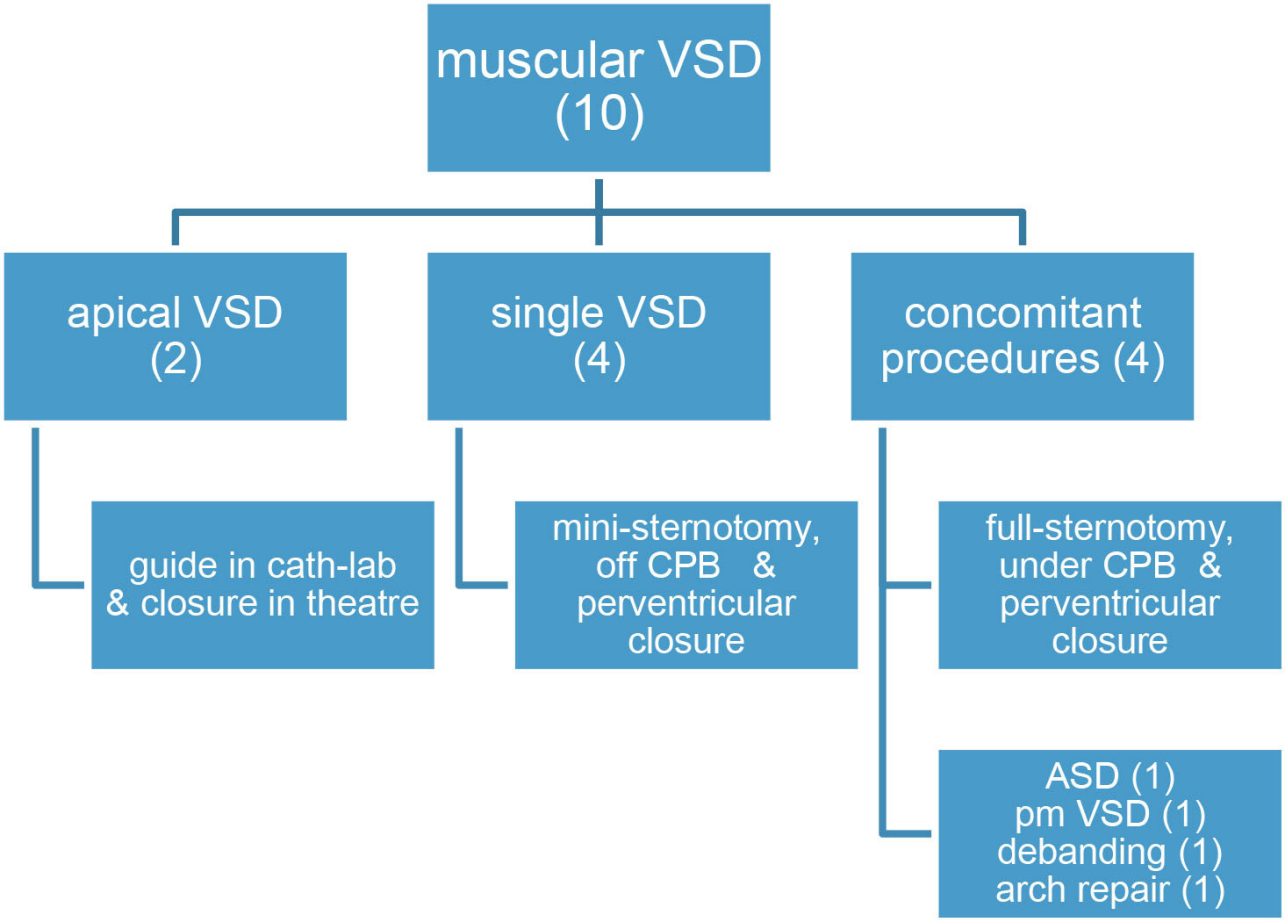 **

## Discussion

In the last 50 years, there has been an incredible evolution and expansion of surgical and percutaneous procedures for patients with congenital heart disease. Hybrid approaches offer the advantages of both disciplines together (17), where neither of them achieve the desired results on their own. A minimally invasive approach can bring substantial benefits on closing a muscular ventricular septal defect in an infant, avoiding cardiopulmonary by-pass and potential vascular complications. This is an example of how to fix a simple defect expeditiously. On the other hand, for those complex cardiac conditions requiring staged surgical and percutaneous procedures, hybrid strategies can spare some of them on addressing surgeon and cardiologist the issue in a collaborative step. The partnership between surgeons and cardiologists will grow as long as the technology develops to catch the new challenges our patients pose.

Since its inception for the palliation of hypoplastic left heart syndrome (1), the concept of hybrid has spread even beyond the univentricular pathway. The potential applications have their boundaries in the collaborative imagination of surgeon and cardiologist, as is shown by the growing number of techniques that are depicted in recent papers (17). Whether to carry out a hybrid procedure in theater or cath-lab can be easily defined (carotid cut-down vs. conduit replacement, e.g.,) but, occasionally, the decision process is far from easy. We arbitrarily agreed to rely on X-ray or cardiopulmonary by-pass needs in order to choose the room. Interestingly, 27 patients in our cohort were hybrid-treated in the cath-lab and the remaining 44 ones were hybrid-operated in theater. Fortunately, the two major complications happened in theater, with a stand-by pump machine to sort them out.

The hybrid procedure is used in neonates with hypoplastic left heart syndrome as an alternative to the conventional Norwood when the risk is considered too high due to prematurity/low weight or other comorbidities, as in our 15 patients. Any comparison between Norwood and hybrid palliation is skewed, since the latter group gathers patients not amenable to Norwood and, hence, at a higher risk (18). Like in other studies (2, 6, 18), around 20–30% (seven out of 15 of our patients) needed a second (or more) visit to the cath-lab for inter-atrial or ductal stenting. Beyond an alternative to Norwood I as a first stage of univentricular repair, the hybrid procedure is emerging as a palliation for other forms of complex left heart lesions so as to increase the chances of a biventricular repair (7, 8), or even a transplant (Table 2). This is the case in our short series, where four out of six patients ended up in biventricular physiology (2 Ross-Konno, 1 Yasui, and 1 arch surgery) and only two in a comprehensive repair (Shone plus mitral stenosis). On the other hand, nine patients were included in the transplant list: seven were successfully transplanted (15, 16) (at 1, 2, 4, 5, 2, 5, and 2 months of life, respectively), and one patient died awaiting a graft (necrotizing enterocolitis, at 5 months). The remaining patient underwent a comprehensive repair at 6 months and 5.4 kg, because a donor was not available before that age. In recent years, we have witnessed a striking decrease in HLHS patients. Our current strategy regards the hybrid procedure as a bridge to transplant in HLHS patients, considering a comprehensive procedure beyond 6 months if not grafted earlier (non published data).

Regarding surgical management of ductal stent at second stage (either comprehensive, biventricular repair—whatsoever—or transplant), several groups have reported their experience in handling the stented ductus/arch (5, 6, 19–22), including deep hypothermia for thorough tissue removal, and complete arch replacement along with heart transplant (15, 16). The prevalence of pulmonary branches intervention at the site of the previous banding is high (5, 6). Whether it is related to the span of time until next stage is accomplished remains to be elucidated, inasmuch prompt comprehensive repair is recommended when intended. We routinely balloon-dilate both pulmonary arteries after band removal since our latest five cases (Yasui, arch repair, three transplants).

According to pulmonary arteries dilatation/stenting along with major surgical repair, our team has gained experience and confidence. Those patients can spare a visit to the cath-lab for branch ballooning/stenting either before or after surgery (10). Interestingly, five children underwent two hybrid procedures at different stages: first, ductal stent plus bilateral banding in the cath-lab and, then, angioplasty/stent of their pulmonary arteries after band removal in theater (three transplants, arch repair, Yasui).

Our experience with Melody device is scarce; just two neonates in the mitral position, and another one in the tricuspid valve, following Boston's report (13). The former patient died shortly after, with a proper function of the prosthesis. The latter underwent a second hybrid approach 2 years later for a valve-in-valve procedure (the Melody regurgitation was likely produced by adherence between one leaflet and the cage). Off-label, we implanted a Melody in pulmonary position concomitant with residual ventricular septal defect repair in two Fallot re-do procedures (Figure 2). The rationale is to provide competence and growth potential in the pulmonary valve. Three years later, both prostheses behave nicely, with no need of dilatation yet.

The hybrid procedure is not complication-free. Learning curve and range of techniques should be born in mind. Two major drawbacks have been recorded. An accidental puncture (Figure 1) in a perventricular approach (9) had no consequences because the use of cardiopulmonary by-pass was scheduled beforehand. The conduit dissection in the sub-xiphoideal approach (12) prompted us to rush and initiate cardiopulmonary by-pass, which was not intended to be used. Fortunately, both unexpected events happened in theater, with a stand-by pump machine and ended up successfully.

Study limitations. The present paper gathers a vast array of congenital conditions and hybrid procedures. Despite arbitrarily clustering the patients in categories alike, the authors are well aware of the difficulties in drawing any early conclusions provided the scarcity of data in every column (Table 1). Authors will expand the scope of the disease thereafter.

## Conclusions

Surgeons and cardiologists alone are often not able to achieve the best therapeutic effect in the field of optography, but the close collaboration between the two may achieve better results in some fields. This study explores both surgeon and cardiologist partnership can succeed where their isolated endeavors are not enough, which provides new ideas and ideas for the treatment of pediatric diseases.

The expectations of the hybrid methods, devised for hypoplastic left heart syndrome, have been overcome by the increasing number of indications which have been successfully applied. Bridging children to biventricular repair or transplant with the hybrid bilateral banding plus ductal stenting is becoming particularly appealing. Patients' challenges and team imagination set the boundaries for this strategy. Equipment, training, and ideas are the basis for innovation, fostering the future of cardiovascular medicine.

On gaining experience, the number of cases and categories will increase providing insight and more robust conclusions.

As a word of caution, hybrid approaches are not complication-free, inasmuch when some procedures are custom-made and tailored to any given patient needs.

## Data Availability Statement

Requests to access the datasets should be directed to the corresponding author at: giljaurena@gmail.com.

## Ethics Statement

The studies involving human participants were reviewed and approved by Hospital Gregorio Marañón's ethics committee. The patients/participants parents provided written informed consent to participate in this study.

## Disclosure

The authors specify that there is no financial, property, or intellectual aid from any commercial source. The authors state that they had full control of the design of the study, methods used, outcome parameters, analysis of data, and production of the written report.

## Author Contributions

All authors contributed to the article and approved the submitted version.

## Conflict of Interest

The authors declare that the research was conducted in the absence of any commercial or financial relationships that could be construed as a potential conflict of interest.

## Publisher's Note

All claims expressed in this article are solely those of the authors and do not necessarily represent those of their affiliated organizations, or those of the publisher, the editors and the reviewers. Any product that may be evaluated in this article, or claim that may be made by its manufacturer, is not guaranteed or endorsed by the publisher.
